# Highly Catalytic Electrochemical Oxidation of Carbon Monoxide on Iridium Nanotubes: Amperometric Sensing of Carbon Monoxide

**DOI:** 10.3390/nano10061140

**Published:** 2020-06-10

**Authors:** Areum Yu, Taehui Kwon, Chongmok Lee, Youngmi Lee

**Affiliations:** Department of Chemistry and Nanoscience, Ewha Womans University, Seoul 03760, Korea; dkfma_5569@nate.com (A.Y.); kwonth9601@gmail.com (T.K.); cmlee@ewha.ac.kr (C.L.)

**Keywords:** iridium, iridium dioxide, nanotubes, carbon monoxide, amperometric sensing, DFT calculation

## Abstract

The nanotubular structures of IrO_2_ and Ir metal were successfully synthesized without any template. First, IrO_2_ nanotubes were prepared by electrospinning and post-calcination, where a fine control of synthetic conditions (e.g., precursor concentration and solvent composition in electrospinning solution, temperature increasing rate for calcination) was required. Then, a further thermal treatment of IrO_2_ nanotubes under hydrogen gas atmosphere produced Ir metal nanotubes. The electroactivity of the resultant Ir metal nanotubes was investigated toward carbon monoxide (CO) oxidation using linear sweep voltammetry (LSV) and amperometry. The anodic current response of Ir metal nanotubes was linearly proportional to CO concentration change, with a high sensitivity and a short response time. The amperometric sensitivity of Ir metal nanotubes for CO sensing was greater than a nanofibrous counterpart (i.e., Ir metal nanofibers) and commercial Pt (20 wt% Pt loading on carbon). Density functional theory calculations support stronger CO adsorption on Ir(111) than Pt(111). This study demonstrates that metallic Ir in a nanotubular structure is a good electrode material for the amperometric sensing of CO.

## 1. Introduction

The production of toxic gases, such as sulfur oxides, nitrogen oxides, hydrocarbons, and carbon monoxide (CO) has risen rapidly in the last few decades. Among them, CO is a colorless and hazardous gas produced as a by-product of fuel combustion, affecting adverse effects on human health as well as the ecosystem [1]. There have been enormous research activities in detection of these harmful gases, which are mainly based on electrochemical (e.g., amperometry and potentiometry) and optical methods (e.g., colorimetry, infrared spectroscopy) [2]. Electrochemical gas sensors have several advantages, including low detection limit, low power consumption, high stability and selectivity [3]. Amperometric gas sensors generally employ a variety of electrode materials catalyzing the electrochemical reactions of target analytes on the electrode surface. In fact, the amperometric sensing of CO requires electrode materials, which facilitate the oxidation of CO and are also highly resistive to the poisoning. In these reasons, various materials (Au [4,5,6], Pt-Sn [7], multi-wall carbon nanotubes [8], Pt covered with Nafion [9], etc. [10,11,12]) have been investigated for the anodic conversion of CO to carbon dioxide (CO_2_), in some cases with water assistance. The mechanism for the CO oxidation in the aqueous acidic condition is shown as follows:

CO + H_2_O → CO_2_ + 2H^+^ + 2e^−^

Elongated nanostructures, including nanofibers, nanorods, nanotubes, and nanobelts, have attracted attention due to their versatile optical, electrical, and mechanical properties [13,14]. Large surface-to-volume ratios and great resistance to agglomeration of these longish nanostructures play important roles in diverse applications. One of the strategies to improve the catalytic efficiencies of a certain material is to enlarge the real surface areas, and thus more active sites can be utilized. In this respect, tubular structures, rather than fibers, are more attractive for the catalytic application [15,16]. Up to date, iridium dioxide (IrO_2_) nanotubes of hollow morphology have been synthesized with diverse methods: e.g., chemical bath deposition using anodic aluminum oxide template [17], electrodeposition in a polycarbonate template [18], metal-organic chemical vapor deposition [19], etc.

In this study, we report the synthesis of IrO_2_ and Ir metal nanotubes without using any templates. IrO_2_ nanotubes are synthesized with electrospinning and subsequent calcination. Thermal annealing of the prepared IrO_2_ nanotubes under H_2_ atmosphere produces Ir metal nanotubes. The resultant Ir metal and IrO_2_ nanotubes are applied for the electrochemical oxidation of CO. The electroactivities of Ir metal or oxide have been previously investigated only for oxygen evolution reaction (OER) [20,21], hydrogen evolution reaction (HER) [22], L-ascorbic acid oxidation [23], but not for CO oxidation. For a clearer understanding of the electrocatalytic CO oxidation at Ir, CO adsorption energy on Ir(111) is also studied theoretically, using density functional theory (DFT) calculation and compared with that on Pt(111).

## 2. Materials and Methods

### 2.1. Materials

Iridium chloride hydrate (IrCl_3_·*x*H_2_O), poly(vinylpyrrolidone) (PVP, MW ≈ 1,300,000), ethanol (99.9%), N,N-dimethylformamide (DMF, anhydrous, 99.8%), sulfuric acid (H_2_SO_4_, 95.0–98.0%) and sodium chloride (NaCl) were all purchased from Sigma-Aldrich (St. Louis, MO, United States). Commercial Pt (cPt, 20 wt% metal loading on Vulcan XC-72) was obtained from Fuel Cell Store (College Station, TX, United States). CO, argon (Ar), helium (He), oxygen (O_2_) and hydrogen (H_2_) gases were obtained from Dong-A Gas Co. (Seoul, Korea). All of the solutions were prepared with deionized water (resistivity ≥ 18 MΩ·cm).

### 2.2. Synthesis of Ir Metal Nanotubes

IrO_2_ nanotubes were synthesized by electrospinning and calcination techniques. Notably, 0.21 g of IrCl_3_·*x*H_2_O was dissolved in 4.5 mL of a mixture of DMF and ethanol (v/v = 3:7) and sonicated for 30 min, then stirred and stabilized overnight. Moreover, 0.3909 g of PVP was added to the solution containing IrCl_3_ and this solution was stirred for 20–24 h to obtain homogeneously mixed solution. Then, the resultant solution was transferred to a syringe with a steel needle (21 GA) and emitted through the needle with a flow rate of 10 μL min^−1^. Furthermore, 13 kV of voltage was applied between the syringe needle and an aluminum plate that are separated by 15 cm using an electrospinning system (NanoNC ESR200R2). The collected electrospun nanofibers were detached from the aluminum plate and dried in a vacuum oven at room temperature to remove the remaining solvents. These as-spun nanofibers were placed in the middle of a furnace. Then, the temperature of the furnace was increased at a rate of 1 °C min^−1^ up to 500 °C, and maintained for 1 h with a flowing of O_2_ and He gases at 10 and 80 sccm, respectively. IrO_2_ nanotubes were eventually obtained after the sufficient cooling at room temperature. These IrO_2_ nanotubes were further heat-treated for the reduction to Ir metal nanofibers, under a continuous flowing of H_2_ and Ar gases at 10 and 80 sccm, respectively. For this process, the temperature of a furnace was raised at a rate of 7.5 °C min^−1^ to reach 250 °C, and maintained for 2 h.

### 2.3. Physical Characterization

The morphologies and structures of IrO_2_ and Ir metal nanotubes were characterized by field-emission scanning electron microscopy (FE-SEM, JEOL JSM-6700F, Tokyo, Japan), high-resolution transmission electron microscopy (HRTEM; JEOL JEM-2100F, Tokyo, Japan), and high-resolution X-ray diffraction (XRD; Rigaku D/Max-2000/PC X-ray diffractometer using Cu Kα radiation, Tokyo, Japan). A thermal analysis of as-spun nanofibers was performed by thermogravimetric analysis and differential scanning calorimetry (TGA-DSC, Q600, TA Instruments, New Castle, DE, United States) to observe the combustion process of PVP. For this measurement, as-spun nanofibers were heated in air from room temperature to 700 °C, at a rate of 5 °C min^−1^.

### 2.4. Electrode Preparation and Electrochemical Measurements

Firstly, synthesized IrO_2_ and Ir metal nanotubes were dispersed in deionized water, with a concentration of 2 mg mL^−1^. A glassy carbon (GC, Austin, TX, United States) electrode (diameter, 3 mm) was loaded with 6 μL of each dispersed solution five times (i.e., 60-μg loading of each material), along with drying in a 60 °C oven between loadings. Then, 10 μL of 0.05 wt% Nafion (diluted in ethanol) was dropped onto the sample loaded GC electrode and dried in air for 30 min. For comparison, 20 μg of cPt was loaded onto the GC electrode with the same procedure.

Electrochemical measurements toward CO oxidation were performed using an electrochemical analyzer (CHI 900B, CH Instrument, Inc., Austin, TX, United States) in 0.5 M H_2_SO_4_, containing 0.03 M NaCl, an optimized condition for CO oxidation [24]. The CO standard solution was prepared by bubbling a deaerated solution of 0.5 M H_2_SO_4_ and 0.03 M NaCl with CO gas for 20 min. A Pt wire and saturated calomel electrode (SCE) were used as the counter and reference electrodes, respectively. Linear sweep voltammetry (LSV) was carried out at various concentrations of CO, with a scan rate of 10 mV·s^−1^. Amperometric response of Ir metal nanotubes to CO concentration changes was measured at 0.7 V (vs. SCE). To prevent CO oxidation through a reaction with O_2_ in the air, a gas-tight cell system was used, and background solutions were deaerated by Ar gas purging. Electrochemical impedance spectroscopy (EIS) was conducted with the CHI 920C electrochemical workstation in 10 mM K_3_Fe(CN)_6_ solution, containing 0.1 M KCl. Cyclic voltammetry (CV) measurements, to investigate the electrochemical surface area (ECSA) of the materials, were conducted in 0.5 M H_2_SO_4_ aqueous solution.

### 2.5. DFT Calculation

DFT calculations were carried out with the generalized gradient approximation (GGA) for the exchange and correlated functional. Vienna ab initio simulation package (VASP), based on the density functional theory (DFT), was used to obtain CO adsorption energy on Ir(111) and Pt(111) by solving the Kohn−Sham equation of a many-body system with an iterative approach. A plane-wave basis set with an adequate cutoff energy of 300 eV and accurate precision was employed. A Monkhorst-Pack mesh of 6 × 6 × 6 *k*-points was used.

In our calculations, the bottom two layers of atoms of the metals were held fixed in their calculated bulk positions, while the upper layers of surface atoms was allowed to move. CO adsorption energy was calculated as below.

*E*_ads_(CO) = *E*_surface-CO,Oa_ − (*E*_surface-Oa_ + *E*_CO_)

where *E*_surface-CO,Oa_, *E*_surface-Oa_, and *E*_CO_ are the total energies of surface adsorbed with both CO and O_a_ (adsorbed oxygen atom), surface adsorbed with O_a_, and CO, respectively.

## 3. Results and Discussion

### 3.1. Synthetic Condition and Physical Characterization of IrO_2_ and Ir Metal Nanotubes

Scheme S1 describes the overall synthetic steps for the nanotubes. Firstly, a homogenously mixed precursor/PVP solution was electrospun to produce nanofibers with a compact body and smooth surface. Obtained as-spun nanofibers were calcined in an atmosphere of O_2_ and He. During this calcination process at 500 °C, IrCl_3_·*x*H_2_O was thermally decomposed to IrO_2_, and PVP playing as framework was removed via the combustion to CO_2_ and H_2_O. Then, the resultant IrO_2_ nanostructures were reduced to form Ir metal counterparts by means of the following reduction process in H_2_ and Ar atmosphere.

**Figure 1:**
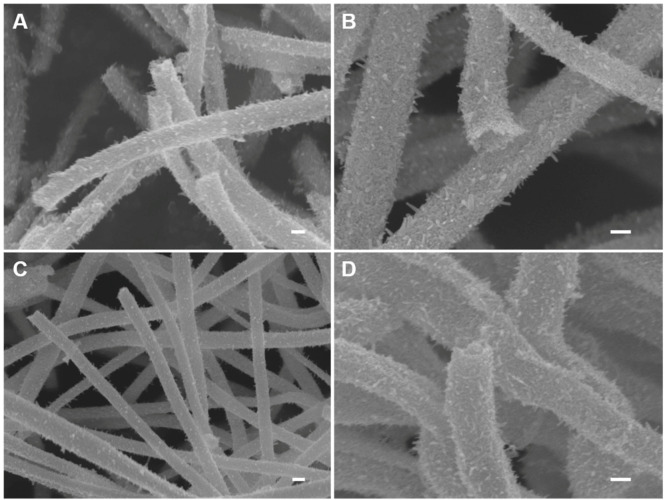
SEM images of (**A**,**B**) IrO_2_ nanotubes and (**C**,**D**) Ir metal nanotubes. (scale bar = 100 nm).

The morphologies of IrO_2_ and Ir metal nanomaterials, before and after the reduction step, respectively, were confirmed by FE-SEM images (Figure 1). The diameters of IrO_2_ and Ir metal nanotubes were estimated to be 200 (±31) nm and 189 (±17) nm, respectively. This implies that the thermal reduction process in the H_2_ atmosphere causes a decrease in diameter. In contrast to the fibrous structure of as-spun IrCl_3_/PVP, both IrO_2_ and Ir metal have tubular morphologies consisting of thorn-sprout outer cylindrical wall and hollow inner space. Tubular morphologies can be built when metal oxides construct rigid walls on the surface of as-spun nanofibers before the complete degradation of PVP [25]. As PVP decomposes into gaseous combustion products (as CO_2_ and H_2_O), the removal of PVP forces metal precursors to move toward the surface. Thus, the degradation of PVP and thermal decomposition of metal precursors are required to occur at properly different temperatures. The precursor concentration and solvent composition in the electrospinning solution also affect the morphology of the spun nanostructures [26,27].

We synthesized elongated IrO_2_ nanostructures under various conditions, of which SEM images were compared in Figure S1. In fact, the IrO_2_ nanotubes shown in Figure 1A,B were synthesized using an electrospinning solution of 0.21 g metal precursor dissolved in a mixed solvent (ethanol/DMF = 70:30 in %v/v), and the applied temperature inclining rate for calcination step was 1 °C·min^−1^. Under the same synthetic condition, except the solvent composition with a lower ethanol content in the electrospinning solution (ethanol/DMF = 50:50 in % v/v), the prepared IrO_2_ appeared to be fibers with a smaller average diameter (Figure S1A) compared to the tubular ones (Figure 1). On the other hand, lowering the amount of metal precursor (0.18 g) in the electrospinning solution also resulted in the fiber form of IrO_2_ as seen in Figure S1B. While holding the other conditions constant, a faster temperature rising rate (3 °C·min^−1^) produced IrO_2_ with fiber morphology as well (Figure S1C). This clearly indicates that the preparation of IrO_2_ nanotubes requires the fine control of the synthetic condition.

**Figure 2:**
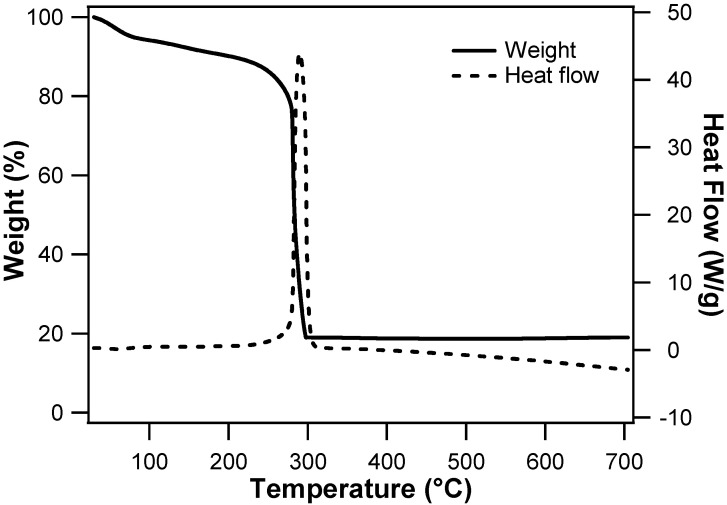
Thermogravimetric analysis and differential scanning calorimetry graph for as-spun IrCl_3_/PVP nanofibers obtained in air, with temperature increasing rate of 5 °C·min^−1^.

For further investigation of the nanotube formation mechanism, TGA-DSC measurements of as-spun nanofibers were conducted (Figure 2). A slight weight decrease around 100 °C can be ascribed to evaporation of solvent and adsorbed water. The following rapid weight loss occurred with a sharp exothermic peak at around 290 °C. The abrupt mass loss between 280 °C and 300 °C is due to the decomposition of IrCl_3_ and PVP; and the weight loss accompanying a sharp exothermic peak at 290 °C is attributed to the oxidation of the main backbone of PVP [25,28]. In fact, the DSC-TGA for pure PVP nanofibers prepared without any metal precursor exhibited a distinct exothermic peak at a higher temperature, ca. 460 °C (data not shown). It has been reported that the presence of metal precursor facilitates the PVP combustion in air [25]. Likewise, the removal of PVP from as-spun IrCl_3_/PVP nanofibers could occur at a lower temperature (ca. 290 °C) than pure PVP with the aid of co-present IrCl_3_. In other words, IrCl_3_ and PVP decompose at quite similar temperatures. Thus, a slow temperature rising rate should be employed for the calcination process, to securely attain a critical condition for nanotube production: metal precursor decomposition, prior to completing PVP removal.

**Figure 3:**
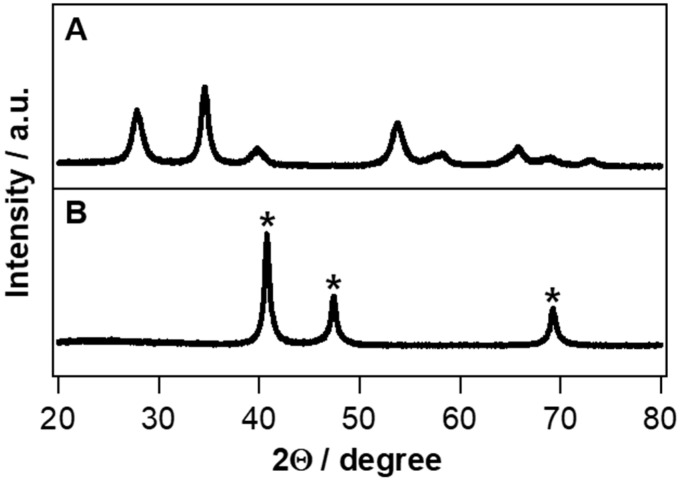
XRD graphs of (**A**) IrO_2_ and (**B**) Ir metal nanotubes. The peaks marked with * are XRD peaks of Ir metal, whereas the peaks without any mark are XRD peaks of IrO_2_.

To investigate the crystal phases, IrO_2_ and Ir metal nanotubes were analyzed with XRD. The XRD spectrum of IrO_2_ showed three major peaks at 27.8°, 34.6° and 53.7°, corresponding to (110), (101) and (211) planes of crystalline IrO_2_ rutile structure, as shown in Figure 3A. After the reduction process in H_2_ atmosphere, the peaks of IrO_2_ rutile structures are not shown. Figure 3B indicates three distinct peaks at 40.7°, 47.4° and 69.2°, being assigned to (111), (200) and (220) planes of the Ir metal fcc crystal structure. This clearly indicates that IrO_2_ has been completely reduced to Ir metal through the reduction process.

The lattice parameter (*a*) for the cubic structure of Ir metal was calculated to be 3.83 Å from the obtained XRD (111) peak, based on Bragg’s law. This will be compared with a spacing of a stabilized Ir metal structure in DFT calculation (*vide supra*). Averaged crystallite sizes of IrO_2_ and Ir metal nanotubes were calculated to be ca. 8.58 and 14.91 nm, respectively, from the major diffraction peaks using the Scherrer formula. The crystallite size of Ir metal nanotubes formed after the thermal reduction was increased compared to IrO_2_ nanotubes, while both the IrO_2_ and Ir metal nanotubes had highly crystalline phases, verified with the sharp XRD peaks.

**Figure 4:**
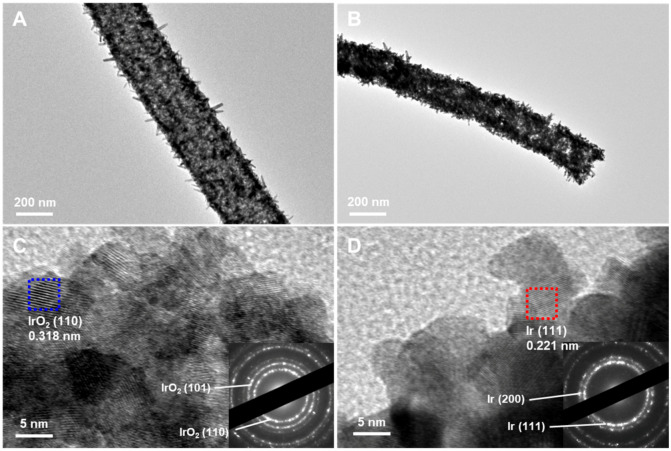
TEM images of (**A**) IrO_2_ and (**B**) Ir metal nanotubes. HRTEM images of (**C**) IrO_2_ and (**D**) Ir metal nanotubes (Insets: SAED patterns).

The tubular morphologies of IrO_2_ and Ir metal nanotubes were confirmed with TEM, as shown in Figure 4. Averaged wall thicknesses of IrO_2_ and Ir metal nanotubes were measured to be 47.1 (±4.7) nm and 49.8 (±4.2) nm, respectively. The crystalline structure of IrO_2_ and Ir metal nanotubes were identified from high-resolution TEM (HRTEM) images. The selected area electron diffraction (SAED) pattern and HRTEM image of IrO_2_ nanotubes clearly indicate the presence of (110) and (101) planes of rutile IrO_2_ phase (Figure 4C). On the other hand, (111) and (200) planes of Ir metal fcc phase were observed in the HRTEM image and SAED pattern of Ir metal nanotubes (Figure 4D). As confirmed by XRD analysis, HRTEM data also support that the nanotubes composed of single phase of IrO_2_ are completely reduced to metallic Ir via a thermal reduction process in the H_2_ atmosphere.

### 3.2. Electrochemical Characterization of the Nanotubes for CO Oxidation

To evaluate the electrochemical properties of the IrO_2_ and Ir metal nanotubes toward CO oxidation, LSVs were performed. To prevent the oxidation of CO via a reaction with O_2_ in the air, a gas-tight cell system was used.

**Figure 5:**
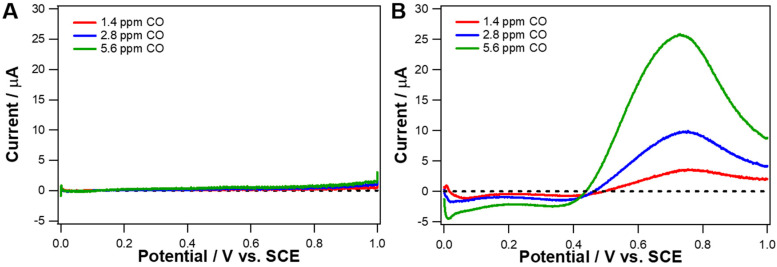
Background-corrected linear sweep voltammetry (LSV) curves of (**A**) IrO_2_ nanotubes- and (**B**) Ir metal nanotubes-loaded glassy carbon (GC) electrode obtained in aqueous solutions at 1.4 ppm (red), 2.8 ppm (blue), and 5.6 ppm (green) of CO concentration. Background solution contains 0.5 M H_2_SO_4_ and 0.03 M NaCl, which is deaerated via Ar gas purging. Scan rate 10 mV s^−1^.

As seen in Figure 5A, the oxidation of CO hardly occurred at IrO_2_ nanotubes within the tested potential range (0.0 to 1.0 V vs. SCE). In contrast, CO oxidation peaks were clearly observed at Ir metal nanotubes, while the peak currents were enhanced with increasing CO concentration (Figure 5B). This implies that electrochemical CO oxidation is more favorable at Ir metal nanotubes than at IrO_2_ nanotubes. Note that cathodic currents were also observed at Ir metal nanotubes for a potential region of 0.0 to 0.4 V (vs. SCE), and these currents increased with CO concentration increase. In fact, cathodic current measured at 0.1 V increased in a linear proportion to CO concentration (Figure S2). This suggests that CO can be reduced at Ir metal nanotubes with a rather low applied potential.

Figure S3 compares the CVs and Nyquist plots of IrO_2_ and Ir metal nanotubes in 10 mM K_3_Fe(CN)_6_ containing 0.1 M KCl. Both IrO_2_ and Ir metal nanotubes showed well-behaved diffusion-controlled CV curves. The charge transfer resistance (R_ct_) values of IrO_2_ nanotubes and Ir metal nanotubes determined from EIS were 83.7 Ω and 97.8 Ω, respectively. Conclusively, both IrO_2_ and Ir metal nanotubes possess low charge transfer resistances.

### 3.3. Amperometric Responses to CO at Ir Metal Nanotubes

**Figure 6:**
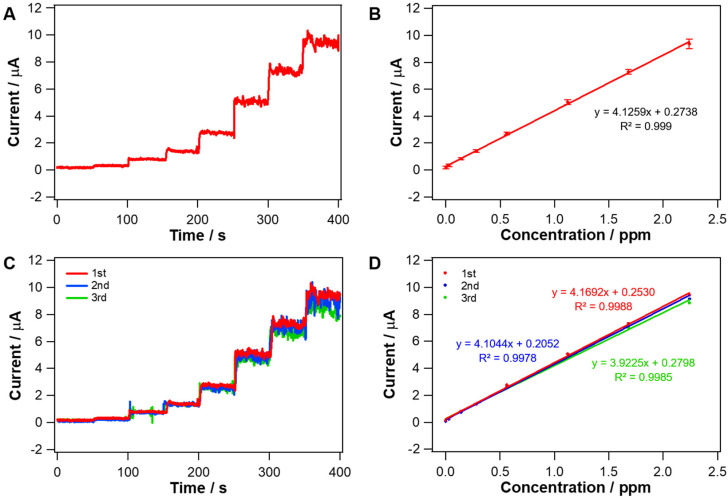
(**A**) Dynamic current response of Ir metal nanotubes-loaded GC electrode to the successive increase of CO concentration (0, 0.028, 0.14, 0.28, 0.56, 1.12, 1.68 and 2.24 ppm), in 0.5 M H_2_SO_4_ aqueous solution containing 0.03 M NaCl. Electrode potential: 0.7 V vs. SCE. (**B**) An averaged calibration curve of Ir metal nanotubes-loaded GC electrodes (*n* = 5) for CO oxidation obtained from dynamic current reponses like (**A**). (**C**) Repeatability test for amperometric responses (three times) on Ir metal nanotubes-loaded GC electrode with an applied electrode potential of 0.7 V vs. SCE. (**D**) Corresponding calibration curves to (**C**).

According to the LSV results shown in Figure 5B, 0.7 V was chosen as an applied potential to monitor the amperometric response to the oxidation of CO. Figure 6A represents the result of the current responding to successive CO injections. Figure 6B illustrates the calibration curves obtained from dynamic current responses (like Figure 6A) of five different electrodes. Small error bars indicate a good reproducibility. Current increased stepwise in a linear proportion to CO concentration (0.028 to 2.24 ppm) in an Ar-saturated aqueous solution, and the sensitivity of Ir metal nanotubes toward CO was 4.1259 μA ppm^−1^ (*n* = 5) with a good linearity of R^2^ > 0.999. In addition, the current of Ir metal nanotubes responded almost immediately to CO injection. The response times (*t*_95%_, time to reach 95% of the steady-state value) were estimated by analyzing amperometric response curves, which were 2.9 ± 0.9 s (*n* = 5). The high sensitivity and linearity of Ir metal nanotubes could be ascribed to both the inherent property of Ir metal (discussed minutely in the DFT section) and the tubular morphology. The large electrochemical active surface area (ECSA) from the nanotube morphology contributes to the high sensitivity towards CO. In Figure 6C, amperometric current responses toward CO were monitored three times for the same concentration range, to test the repeatability and resistivity to the surface contamination. The sensitivities toward CO were nearly unchanged for the repeated measurements (Figure 6D). It indicates that Ir metal nanotubes were stable enough without surface fouling during CO oxidation.

**Figure 7:**
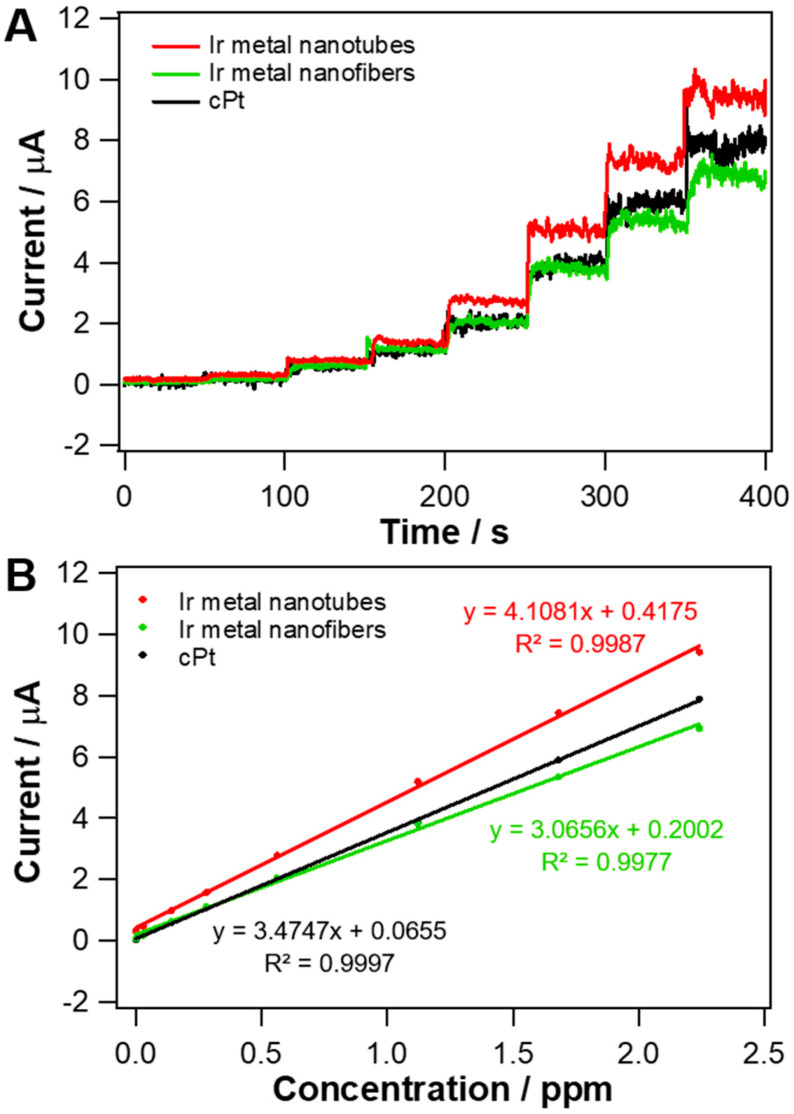
(**A**) Dynamic current responses of cPt, Ir metal nanofibers and Ir metal nanotubes-loaded GC electrode to the successive increase of CO concentration (0, 0.028, 0.14, 0.28, 0.56, 1.12, 1.68 and 2.24 ppm), in 0.5 M H_2_SO_4_ aqueous solution containing 0.03 M NaCl. Electrode potential: 0.7 V vs. SCE. (**B**) Corresponding calibration curves to (**A**).

Amperometric response and sensitivity for CO oxidation were also measured at Ir metal nanofibers and cPt, and compared with those obtained at Ir metal nanotubes (Figure 7). Ir metal nanofibers used for this experiment were the ones formed by the reduction of IrO_2_ nanofibers synthesized with a different solvent composition (ethanol/DMF = 50:50 in % v/v), shown in Figure S1A. As clearly seen in Figure 7B, the sensitivity of Ir metal nanotubes for amperometric CO sensing was 1.34-fold greater than that of Ir metal nanofibers, having the same composition but a tubular structure. Figure S4 represents CV curves of Ir metal nanotubes and Ir metal nanofibers obtained in 0.5 M H_2_SO_4_. The electrochemical surface area (ECSA) of each material was estimated using the charge amount for the hydrogen adsorption region in CV curves and the specific charge value for Ir metal (218 μC·cm^−2^) [29]. The ECSAs of Ir metal nanotubes and nanofibers were determined to be 8.29 cm^2^ and 6.35 cm^2^, respectively. Ir metal nanotubes had ca. 1.31-fold larger ECSA than Ir metal nanofibers. This clearly suggests that a tubular structure providing a larger surface area is advantageous over a fibrous one for catalyzing CO oxidation. Ir metal nanotubes also exhibited ~20% higher sensitivity for CO oxidation than cPt, a most commonly used heterogenous electrocatalyst, supporting its high electroactivity. Note that an applied potential of 0.7 V was also sufficient for CO oxidation at cPt, which was confirmed with LSV (Figure S5).

A continuous amperometric measurement of CO was carried out with Ir metal nanotubes at 0.7 V (vs. SCE) in 0.5 M H_2_SO_4_ containing 0.03 M NaCl aqueous solution, being purged with CO gas (Figure S6). Ir metal nanotubes maintained 97.6 % of the initial CO oxidation current after a 5000-s continuous measurement, indicating a reasonable stability (Figure S6).

The amperometric CO sensing performance of Ir metal nanotubes was compared with the previously reported materials in Table S1. Compared to other reported catalysts, Ir metal nanotubes were found to possess much higher sensitivity (normalized to geometric surface area) and lower detection limit, being advantageous particularly in the measurements of relatively low CO levels. Therefore, these materials have potential applications for monitoring air contamination by CO generated from the incomplete combustion of carbon-based fuels with high sensitivity and low detection limit.

### 3.4. DFT Calculations for the CO Adsorption Energy

The interaction between reactants and catalyst surfaces is an important step in most heterogeneous catalytic reactions, such as CO oxidation and hydrogenation [30]. CO adsorption energy on Ir(111) was calculated by means of DFT and compared with that on Pt(111), which has been known as an excellent CO oxidation catalyst [31,32]. Before introducing CO and O_a_, the structural optimizations for Pt(111) and Ir(111) were carried out. With the optimized metal structures, the lattice parameters were obtained to be 3.87 Å and 3.96 Å for Ir(111) and Pt(111), respectively. The calculated Ir metal lattice parameter, 3.87 Å, was very similar to the value (3.83 Å) estimated from XRD results, as shown in Figure 3B, indicating the reliability of our calculation.

**Figure 8:**
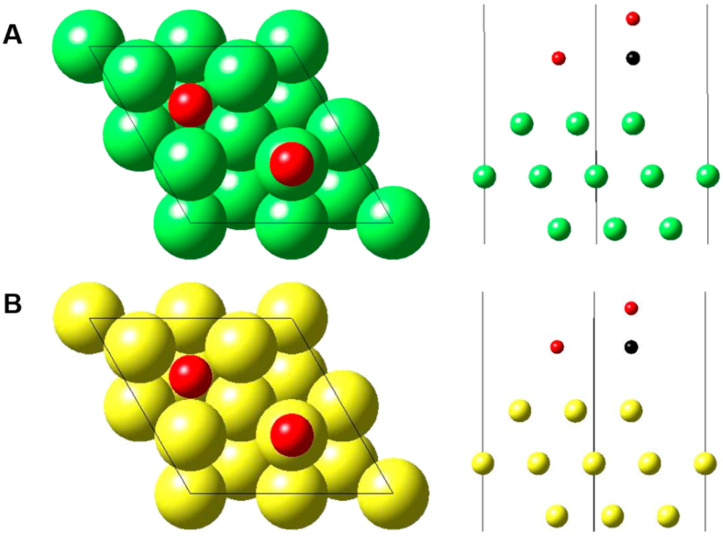
Illustration of CO and O_a_ adsorption on (**A**) Ir(111) and (**B**) Pt(111) surfaces. Ir, Pt, O and C atoms are represented in green, yellow, red and black, respectively.

A previous study suggested that CO preferentially adsorbs on the atop sites of Pt and Ir [30,33,34]. Adsorbed oxygen atom (denoted as O_a_) was introduced with CO concurrently at the surface for DFT calculation [34]. The adsorption of oxygen is necessary to oxidize CO to CO_2_, and O_a_ could be from metal surface native oxide or any oxygen containing species near the surface. In our calculation, a CO molecule was also found to adsorb on atop sites of both Ir and Pt atoms through the carbon atom, while O_a_ sat on the fcc hollow site of three atoms. The calculated structures are shown in Figure 8.

The calculated adsorption energies of CO on Ir(111) and Pt(111) surfaces were 2.02 and 1.54 eV, respectively, demonstrating a stronger CO adsorption on Ir(111) than on Pt(111). Thus, the observed greater CO sensitivity of Ir metal nanotubes could be attributed to a facile CO adsorption on the Ir metal surface. However, the nanofibers of Ir metal showed a similar/slightly lower CO sensitivity compared with cPt. This implies that metallic Ir has an inherent good electroactivity for CO electrochemical oxidation, but morphology control is also another important factor for efficient application.

## 4. Conclusions

IrO_2_ nanotubes were fabricated by electrospinning and subsequent calcination in the O_2_ atmosphere. The production of nanotubular morphology of IrO_2_ necessitates the optimization of electrospinning solution composition (e.g., concentration and solvent composition) and temperature rising rate for the calcination step. The prepared IrO_2_ nanotubes were further reduced to Ir metal nanotubes under the thermal reduction process in H_2_ atmosphere. The resultant Ir metal nanotubes showed an excellent electroactivity for electrochemical CO oxidation; and the dynamic current response at a constant applied potential of 0.7 V vs. SCE increased in a linear proportion to CO concentration change, with a reasonable short response time. The CO sensitivity of nanotubular Ir metal was ca. 35% higher than a nanofibrous counterpart (i.e., Ir metal nanofibers) and even better than commercial Pt (20 wt% Pt loading on carbon). DFT calculation showed that Ir(111) has higher adsorption energy for CO than Pt(111). Conclusively, the enlarged surface area of a tubular morphology and the inherent property of metallic Ir for facile CO adsorption synergistically present Ir metal nanotubes as a good electrode material for amperometric CO sensing.

## Supplementary Materials

The following are available online at https://www.mdpi.com/2079-4991/10/6/1140/s1, Scheme S1: synthetic scheme, Figure S1: SEM images, Figure S2: Dynamic current response for CO reduction, Figure S3: CV and EIS data obtained in 10 mM K_3_Fe(CN)_6_ containing 0.1 M KCl, Figure S4: CV in 0.5 M H_2_SO_4_, Figure S5: LSV results of cPt for CO oxidation, Figure S6: Chronoamperometric response for 5 000 s, Table S1: Comparison table.

## References

[1] Jin Y., Guo L., Veiga M.C., Kennes C. (2009). Optimization of the treatment of carbon monoxide-polluted air in biofilters. Chemosphere.

[2] Yunusa Z., Hamidon M.N., Kaiser A., Awang Z. (2014). Gas sensors: A review. Sens. Transducers J..

[3] Van der Wal P.D., de Rooij N.F., Koudelka-Hep M. (1996). Extremely stable nafion based carbon monoxide sensor. Sens. Actuators B.

[4] Chen C., He J., Xu D., Tan X., Zhou X., Wang X. (2005). Study of nano-Au-assembled amperometric CO gas sensor. Sens. Actuators B.

[5] Rodriguez P., Koper M.T.M. (2014). Electrocatalysis on gold. Phys. Chem. Chem. Phys..

[6] Hayden B.E., Pletcher D., Suchsland J.-P. (2007). Enhanced activity for electrocatalytic oxidation of carbon monoxide on titania-supported gold nanoparticles. Angew. Chem. Int. Ed..

[7] Wang K., Gasteiger H.A., Markovic N.M., Ross P.N. (1996). On the reaction pathway for methanol and carbon monoxide electrooxidation on Pt-Sn alloy versus Pt-Ru alloy surfaces. Electrochim. Acta.

[8] Santhosh P., Manesh K.M., Gopalan A., Lee K.-P. (2007). Novel amperometric carbon monoxide sensor based on multi-wall carbon nanotubes grafted with polydiphenylamine—Fabrication and performance. Sens. Actuators B.

[9] Yasuda A., Shimidzu T. (1999). Electrochemical carbon monoxide sensor with a Nafion^®^ film. React. Funct. Polym..

[10] Li X., Xuan T., Yin G., Gao Z., Zhao K., Yan P., He D. (2015). Highly sensitive amperometric CO sensor using nanocomposite C-loaded PdCl_2_–CuCl_2_ as sensing electrode materials. J. Alloys Compd..

[11] Tsceng K.-I., Yang M.-C. (2003). Platinum electrodes modified by tin for electrochemical CO sensors. J. Electrochem. Soc..

[12] Guan Y., Liu F., Wang B., Yang X., Liang X., Suo H., Sun P., Sun Y., Ma J., Zheng J. (2017). Highly sensitive amperometric Nafion-based CO sensor using Pt/C electrodes with different kinds of carbon materials. Sens. Actuators B.

[13] Xia Y., Yang P., Sun Y., Wu Y., Mayers B., Gates B., Yin Y., Kim F., Yan H. (2003). One-dimensional nanostructures: Synthesis, characterization, and applications. Adv. Mater..

[14] Liang H.-W., Liu S., Yu S.-H. (2010). Controlled synthesis of one-dimensional inorganic nanostructures using pre-existing one-dimensional nanostructures as templates. Adv. Mater..

[15] Chu Z., Cheng H., Xie W., Sun L. (2012). Effects of diameter and hollow structure on the microwave absorption properties of short carbon fibers. Ceram. Int..

[16] Homaeigohar S., Davoudpour Y., Habibi Y., Elbahri M. (2017). The electrospun ceramic hollow nanofibers. Nanomaterials.

[17] Chen P.-C., Chen Y.-C., Huang C.-N. (2018). Free-standing iridium oxide nanotube array for neural interface electrode applications. Mater. Lett..

[18] Mafakheri E., Salimi A., Hallaj R., Ramazani A., Kashi M.A. (2011). Synthesis of iridium oxide nanotubes by electrodeposition into polycarbonate template: Fabrication of chromium(III) and arsenic(III) electrochemical sensor. Electroanalysis.

[19] Chen R.-S., Huang Y.-S., Tsai D.-S., Chattopadhyay S., Wu C.-T., Lan Z.-H., Chen K.-H. (2004). Growth of well aligned IrO_2_ nanotubes on LiTaO_3_(012) substrate. Chem. Mater..

[20] Geiger S., Kasian O., Shrestha B.R., Mingers A.M., Mayrhofer K.J.J., Cherevko S. (2016). Activity and stability of electrochemically and thermally treated iridium for the oxygen evolution reaction. J. Electrochem. Soc..

[21] Antolini E. (2014). Iridium as catalyst and cocatalyst for oxygen evolution/reduction in acidic polymer electrolyte membrane electrolyzers and fuel cells. ACS Catal..

[22] Kim S.-J., Jung H., Lee C., Kim M.H., Lee Y. (2019). Comparative study on hydrogen evolution reaction activity of electrospun nanofibers with diverse metallic Ir and IrO_2_ composition ratios. ACS Sustain. Chem. Eng..

[23] Kim S.-J., Kim Y.L., Yu A., Lee J., Lee S.C., Lee C., Kim M.H., Lee Y. (2014). Electrospun iridium oxide nanofibers for direct selective electrochemical detection of ascorbic acid. Sens. Actuators B.

[24] Lee Y., Kim J. (2007). Simultaneous electrochemical detection of nitric oxide and carbon monoxide generated from mouse kidney organ tissues. Anal. Chem..

[25] Zhang G., Wang Y., Wang X., Chen Y., Zhou Y., Tang Y., Lu L., Bao J., Lu T. (2011). Preparation of Pd–Au/C catalysts with different alloying degree and their electrocatalytic performance for formic acid oxidation. Appl. Catal. B.

[26] Moon S., Cho Y.-B., Yu A., Kim M.H., Lee C., Lee Y. (2019). Single-step electrospun Ir/IrO_2_ nanofibrous structures decorated with Au nanoparticles for highly catalytic oxygen evolution reaction. ACS Appl. Mater. Interfaces.

[27] Li L., Peng S., Cheah Y., Teh P., Wang J., Wee G., Ko Y., Wong C., Srinivasan M. (2013). Electrospun porous NiCo_2_O_4_ nanotubes as advanced electrodes for electrochemical capacitors. Chem. Eur. J..

[28] Kim S.Y., Yu A., Lee Y., Kim H.Y., Kim Y.J., Lee N.-S., Lee C., Lee Y., Kim M.H. (2019). Single phase of spinel Co_2_RhO_4_ nanotubes with remarkably enhanced catalytic performance for the oxygen evolution reaction. Nanoscale.

[29] Łukaszewski M., Soszko M., Czerwiński A. (2016). Electrochemical methods of real surface area determination of noble metal electrodes—An overview. Int. J. Electrochem. Sci..

[30] Orita H., Itoh N., Inada Y. (2004). All electron scalar relativistic calculations on adsorption of CO on Pt(111) with full-geometry optimization: A correct estimation for CO site-preference. Chem. Phys. Lett..

[31] Allian A.D., Takanabe K., Fujdala K.L., Hao X., Truex T.J., Cai J., Buda C., Neurock M., Iglesia E. (2011). Chemisorption of CO and mechanism of CO oxidation on supported platinum nanoclusters. J. Am. Chem. Soc..

[32] McPherson I.J., Ash P.A., Jones L., Varambhia A., Jacobs R.M.J., Vincent K.A. (2017). Electrochemical CO oxidation at platinum on carbon studied through analysis of anomalous in situ IR spectra. J. Phys. Chem. C.

[33] Zou S., Gómez R., Weaver M.J. (1997). Nitric oxide and carbon monoxide adsorption on polycrystalline iridium electrodes:  A combined raman and infrared spectroscopic study. Langmuir.

[34] Zhang C.J., Baxter R.J., Hu P., Alavi A., Lee M.H. (2001). A density functional theory study of carbon monoxide oxidation on the Cu_3_Pt(111) alloy surface: Comparison with the reactions on Pt(111) and Cu(111). J. Chem. Phys..

