# Supplementary material for: Highly Catalytic Electrochemical Oxidation of Carbon Monoxide on Iridium Nanotubes: Amperometric Sensing of Carbon Monoxide
Source: Nanomaterials (Basel). 2020 Jun 10;10(6):1140. doi: 10.3390/nano10061140 (PMC7353436; doi:10.3390/nano10061140)
Supplement: Supplementary file 1 [file nanomaterials-10-01140-s001.docx]

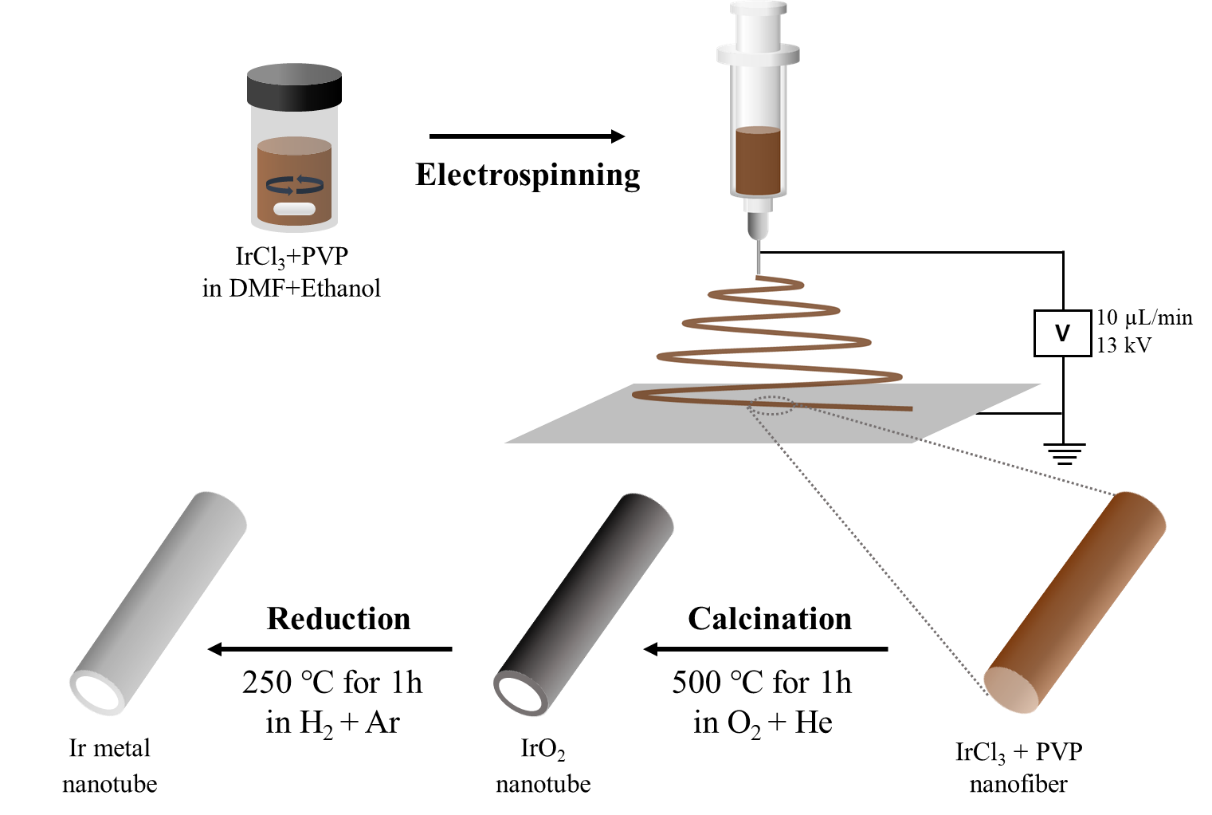


**Scheme S1.** Synthetic scheme of Ir metal nanotubes.


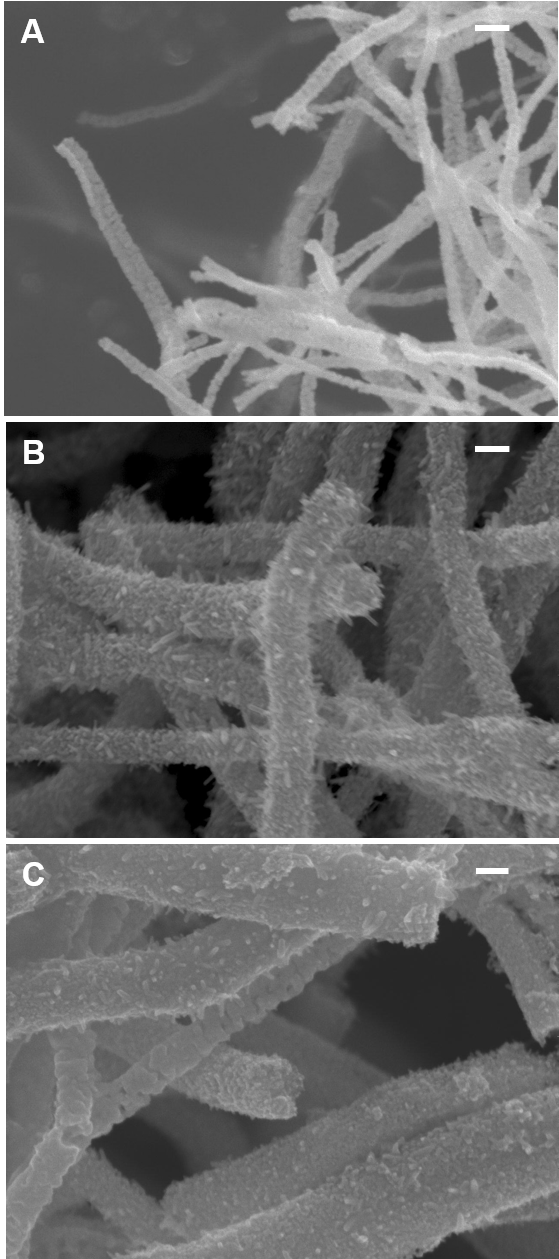


**Figure S1.** SEM images of differently synthesized IrO_2_ nanostructures. The corresponding synthetic conditions of solvent composition ratio (ethanol:DMF), metal precursor weight in electrospinning solution and temperature increasing rate are (**A**) **5:5**, 0.21 g and 1 °C min^−1^, (**B**) 7:3, **0.18 g**, 1 °C min^−1^, and (**C**) 7:3, 0.21 g, **3 °C min^−1^**. Scale bar = 100 nm. The different synthetic factors from the ones shown in Figure 1 are marked in bold.


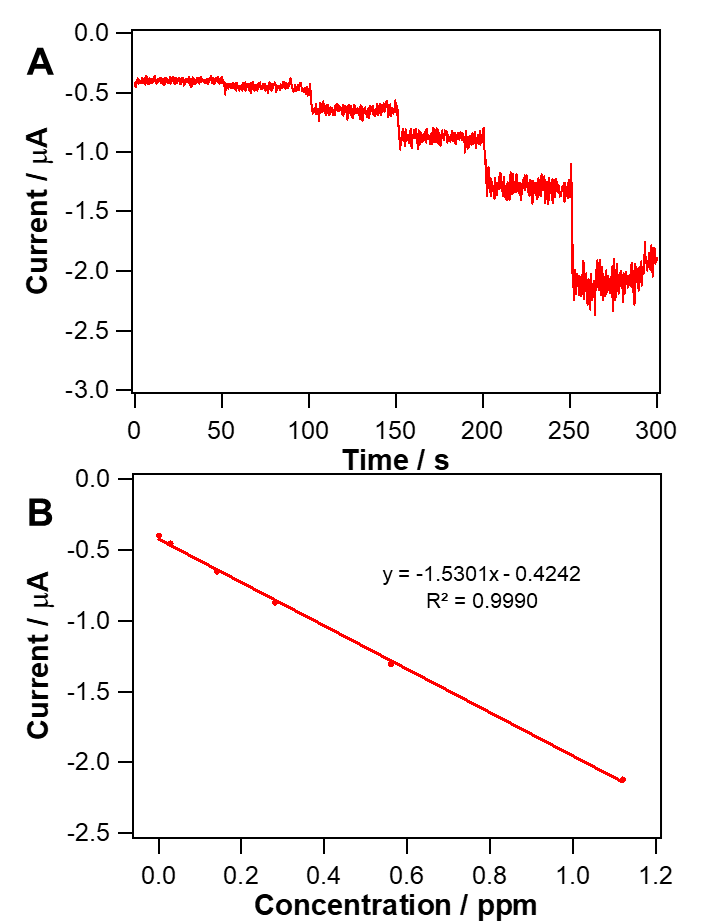


**Figure S2.** (**A**) Dynamic current response of Ir metal nanotube-loaded GC electrode to the successive increases of CO concentration (0, 0.028, 0.14, 0.28, 0.56 and 1.12 ppm) in 0.5 M H_2_SO_4_ aqueous solution containing 0.03 M NaCl. Electrode potential: 0.1 V vs SCE. (**B**) Corresponding calibration curve for CO reduction.


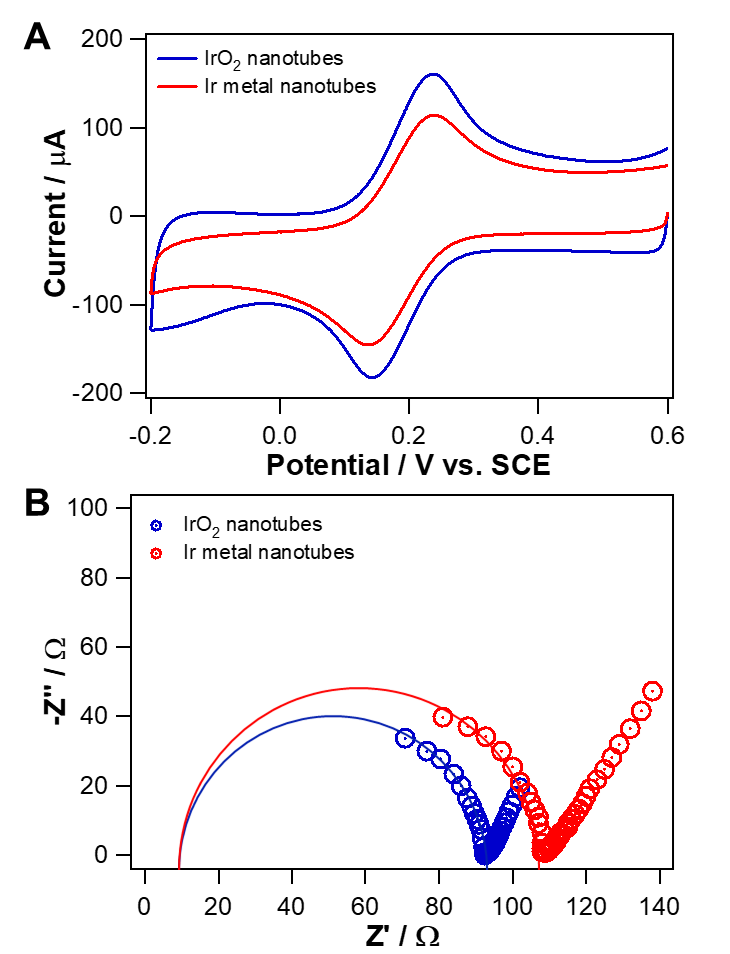


**Figure S3.** (**A**) Cyclic voltammograms at 50 mV s^−1^ and (**B**) Nyquist plots of IrO_2_ nanotubes and Ir metal nanotubes-loaded GC electrode in 10 mM K_3_Fe(CN)_6_ containing 0.1 M KCl. Nyquist plots are measured at cathodic peak potential in the frequency of 10 Hz to 1 MHz.

**Figure S4.** Cyclic voltammograms (CVs) of Ir metal nanotubes and Ir metal nanofibers in 0.5 M H_2_SO_4_ at 20 mV s^−1^.

**Figure S5.** Background-corrected LSV curves of cPt-loaded GC electrode obtained in aqueous solutions at 1.4 ppm (red), 2.8 ppm (blue), and 5.6 ppm (green) of CO concentration. Background solution contains 0.5 M H_2_SO_4_ and 0.03 M NaCl which is deaerated via Ar gas purging. Scan rate 10 mV s^−1^.

**Figure S6.** The chronoamperometric response of Ir metal nanotubes for the CO oxidation for 5 000 s with an applied potential of 0.7 V (vs SCE) in CO-saturated 0.5 M H_2_SO_4_ aqueous solution containing 0.03 M NaCl.

**Table S1.** Comparison of the catalytic performance toward CO oxidation of Ir metal nanotubes with other CO oxidation catalysts.

| **Sample** | **Electrolytes** | **Potential** | **Sensitivity** | **LOD**  **(ppm)** | **Linear range**  **(ppm)** | **Ref** |
| --- | --- | --- | --- | --- | --- | --- |
| Ir metal nanotubes | 0.5 M H_2_SO_4_  + 0.03 M NaCl | 0.7 V  (vs SCE) | 51.57*^a^* | 0.018 | 0.028 - 2.24 | This work |
| Nano-Au modified Pt disk electrode | 0.5 M HClO_4_ | 0.8 V (vs Ag/AgCl) | 1.82$\times$10^−1^ *^a^* | 650 | 700 - 56,000 | [4] |
| C-loaded PdCl_2_–CuCl_2_ | Nafion | 0.4 V  (vs. nano-Pt powder) | 2.69 *^a^* | 1 | 1 - 100 | [10] |
| Sn/Pt/Nafion assembly | 0.5 M H_2_SO_4_ | 0.5 V (vs. Ag/AgCl) | 1.26$\times$10^−1^ *^a^* | - | 0 - 400 | [11] |
| Pt NPs supported on CFs | Nafion | - (vs Pt loading) | 0.077 *^b^* | 0.1 | 1 - 200 | [12] |

Units of sensitivities are *^a^*μA ppm^−1^cm^−2^ and *^b^*μA ppm^−1.^
